# A Transfer Learning Framework for Predicting and Interpreting Drug Responses via Single-Cell RNA-Seq Data

**DOI:** 10.3390/ijms26094365

**Published:** 2025-05-04

**Authors:** Yujie He, Shenghao Li, Hao Lan, Wulin Long, Shengqiu Zhai, Menglong Li, Zhining Wen

**Affiliations:** 1 College of Chemistry, Sichuan University, Chengdu 610064, China; he_yujie@stu.scu.edu.cn (Y.H.);; 2 Medical Big Data Center, Sichuan University, Chengdu 610064, China

**Keywords:** drug response, single-cell RNA sequencing, bulk RNA sequencing, deep learning, interpretability

## Abstract

Chemotherapy is a fundamental therapy in cancer treatment, yet its effectiveness is often undermined by drug resistance. Understanding the molecular mechanisms underlying drug response remains a major challenge due to tumor heterogeneity, complex cellular interactions, and limited access to clinical samples, which also hinder the performance and interpretability of existing predictive models. Meanwhile, single-cell RNA sequencing (scRNA-seq) has emerged as a powerful tool for uncovering resistance mechanisms, but the systematic collection and utilization of scRNA-seq drug response data remain limited. In this study, we collected scRNA-seq drug response datasets from publicly available web sources and proposed a transfer learning–based framework to align bulk and single cell sequencing data. A shared encoder was designed to project both bulk and single-cell sequencing data into a unified latent space for drug response prediction, while a sparse decoder guided by prior biological knowledge enhanced interpretability by mapping latent features to predefined pathways. The proposed model achieved superior performance across five curated scRNA-seq datasets and yielded biologically meaningful insights through integrated gradient analysis. This work demonstrates the potential of deep learning to advance drug response prediction and underscores the value of scRNA-seq data in supporting related research.

## 1. Introduction

Chemotherapy remains one of the core therapeutic strategies in modern medicine and is widely applied across a range of diseases, from common infections to complex conditions. Among these, cancer is one of the most prominent diseases for which chemotherapy plays a central role in treatment. Although chemotherapy is widely regarded as an effective approach for cancer management and often produces substantial initial responses, therapeutic failure frequently occurs due to tumor cell adaptation to pharmacological agents, ultimately leading to the development of drug resistance [1,2]. The mechanisms of chemoresistance are complex, involving tumor cell heterogeneity, interactions within the tumor microenvironment, and dysregulation of signaling pathways [3,4,5,6]. For instance, activation of the NF-κB, STAT3, and PI3K pathways has been widely implicated in causing chemoresistance during cancer therapy [7,8,9]. Consequently, elucidating the molecular basis of drug resistance holds critical importance for developing novel anticancer therapeutics and optimizing clinical treatment strategies [10,11]. However, research on drug response faces challenges. Investigating the biological mechanisms of drug responses at the molecular and cellular levels is inherently difficult. Drug resistance is highly complex and varies significantly across individuals, tissue types, and drugs. Furthermore, the limited availability to obtain samples from clinical trials constrains drug response studies [12,13,14].

To address these issues, early computational approaches incorporated genomic features, such as somatic mutations and copy number variations, along with pharmacochemical properties like molecular fingerprints, to develop machine learning (ML) models, including random forests (RFs) and shallow neural networks. While these models achieved promising results, they often struggled with high-dimensional omics data and lacked interpretability, limiting their ability to reveal the biological basis of predictions [15].

Recent advances in deep learning (DL) and high-throughput screening technologies have catalyzed developments in drug response prediction [16]. DL models can automatically extract complex features from large-scale biological datasets, enabling more accurate prediction. The increasing availability of data resources has further driven the systematic integration of DL into pharmacogenomic modeling, resulting in a shift from simplistic statistical frameworks to sophisticated deep learning architectures.

MOLI [17] utilized independent encoders to separately extract features from somatic mutations, copy number variations, and gene expression profiles, which were subsequently concatenated and input into a neural network for drug response prediction. DeepDRK [18] employed kernel functions to extract features from cellular omics and drug data, which are fused as inputs. CODE-AE-ADV [19] adopted a deconfounding adversarial autoencoder to learn robust latent representations that align in vitro with in vivo data. In addition to performance improvements, several DL models aim to enhance interpretability. DrugCell [20] integrated a visible neural network (VNN), aligning model architectures with tumor cellular organizations to simulate therapeutic mechanisms. ParsVNN [21] enhanced performance and interpretability through pruning VNN, streamlining biological hierarchies to focus on critical features. Moreover, advanced neural architectures such as convolutional neural networks, graph neural networks, and transformer-based models have also been explored for modeling cellular omics and drug response, yielding promising results [22,23,24].

Meanwhile, single-cell RNA sequencing (scRNA-seq) has emerged as a transformative approach for dissecting tumor heterogeneity and elucidating mechanisms of drug response [25]. Compared with bulk RNA sequencing (bulk RNA-seq), scRNA-seq enables the identification of differences among distinct cellular subpopulations and capture subtle molecular changes, making it more sensitive for predicting drug responses [25,26]. As a result, it can provide a more precise understanding of the mechanisms driving drug responses. Despite these advantages, the majority of drug response models still rely on bulk RNA-seq data, such as Genomics of Drug Sensitivity in Cancer (GDSC) [27] and L1000 [28]. Although several scRNA-seq datasets, such as MIX-seq [29] and sci-Plex [30], have recently been introduced, systematic collection and analysis of scRNA-seq drug response data remain limited. This is largely due to the relatively high cost of scRNA-seq experiments, as well as the time-consuming and labor-intensive process of manual data annotation [31,32]. The limited data hinders accurate prediction and in-depth understanding of mechanisms driving drug response. In contrast, large-scale bulk RNA-seq drug response datasets are already available. Leveraging these abundant datasets to train DL models, which is a data-driven method, and applying transfer learning to adapt the models to single-cell RNA-seq data for accurate prediction and mechanistic investigation, offers a promising strategy to partially overcome the current limitations caused by the scarcity of high-quality scRNA-seq drug response data.

**Figure 1:**
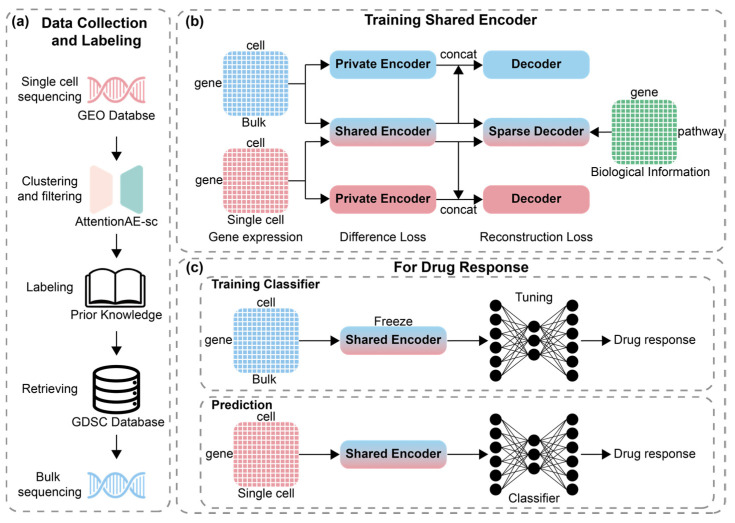
Overview of data processing and model construction. (**a**) Pipeline of data collection and labeling; (**b**) pretraining for autoencoder with both sequencing data; (**c**) training classifier and prediction for drug response with pretrained shared encoder.

This study systematically processed scRNA-seq drug response data and proposed a transfer learning–based framework to align drug response representations between bulk RNA-seq and scRNA-seq data (Figure 1). Specifically, we leveraged the GDSC dataset, which is a bulk RNA-seq dataset, to train a predictive model for scRNA-seq drug response. This model employed a shared encoder to project both data into a unified latent space, and the projected embeddings were utilized to predict drug responses. A sparse decoder guided by prior biological knowledge was integrated to align features with predefined biological pathways for enhancing performance and interpretability. The proposed model was evaluated across five curated scRNA-seq drug response datasets and demonstrated superior predictive performance compared to ML models. Meanwhile, we applied integrated gradients (IG) to interpret the relationship between pathways and drug response predictions. Also, we validated interpretability based on the biological information introduced via the sparse decoder. This study contributes to expanding the utility of scRNA-seq in drug response prediction and the application of DL techniques in drug response prediction.

## 2. Results

### 2.1. Data Analysis and Clustering Analysis

Details of data collection and labeling were described in Figure 1a. The five collected scRNA-seq datasets involved three cancers, including oral squamous cell carcinoma (GSE117872), melanoma (GSE108394), and breast cancer (GSE131984, GSE156246_BT474, and GSE156246_HCC1419). These datasets involved four drugs (cisplatin, paclitaxel, PLX-4720, and lapatinib) including two chemotherapeutic agents and two targeted therapies, as shown in Table 1. More datasets that do not have corresponding entries in the GDSC database can be found in Supplementary Material S1.

We retrieved corresponding drug response data for these four drugs from the GDSC database (bulk RNA-seq) to construct training sets for predicting drug response. In the drug response dataset of GDSC, the number of cell lines tested for each drug varies, typically ranging from 735 to 903. Datasets include half maximal inhibitory concentration (IC50) (lower values indicate stronger drug efficacy and higher sensitivity) and the area under the dose–response curve (AUC) (lower values indicate higher drug sensitivity). In this study, we used the AUC value as the indicator of drug response. It was employed to define positive and negative samples in the training sets and to train four corresponding binary classifiers of drug response for four drugs, respectively.

We then performed clustering analysis on the collected five collected scRNA-seq drug response datasets with AttentionAE-sc [33], which is our previously proposed method. The clustering results were used to distinguish drug-sensitive and drug-resistant cell subpopulations, serving as a basis for data filtering. To quantitatively evaluate the clustering performance, we computed the average silhouette width (ASW) as an internal validation metric. A higher ASW score indicates better clustering quality, characterized by greater separation between clusters and higher compactness within clusters. Additionally, we visualized the clustering results via Uniform Manifold Approximation and Projection (UMAP).

**Figure 2:**
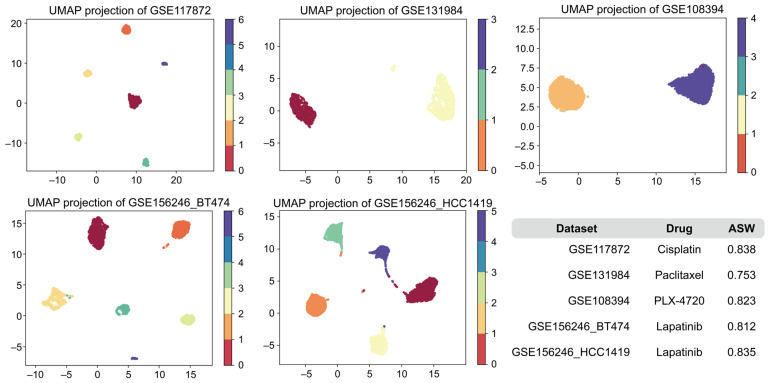
UMAP visualization of clustering results for scRNA-seq drug response datasets. UMAP: Uniform Manifold Approximation and Projection; ASW: Average silhouette width.

The results are shown in Figure 2. Across all datasets, the model achieved ASW scores above 0.75. Specifically, four datasets had ASW scores greater than 0.8, reflecting well-separated and internally coherent clusters. These results suggest that the model effectively captures meaningful transcriptional differences between drug-sensitive and drug-resistant subpopulations. This provides a strong foundation for subsequent drug response labeling and downstream prediction tasks. A comparison with other clustering methods is provided in Supplementary Material S2.

### 2.2. Performance Evaluation

To evaluate the performance of our model on scRNA-deq test sets, we compared it with several widely used ML algorithms. These included logistic regression (LR), support vector machine (SVM), decision tree (DT), RF, and gradient boosting (GB) and eXtreme Gradient Boosting (XGBoost.)

**Figure 3:**
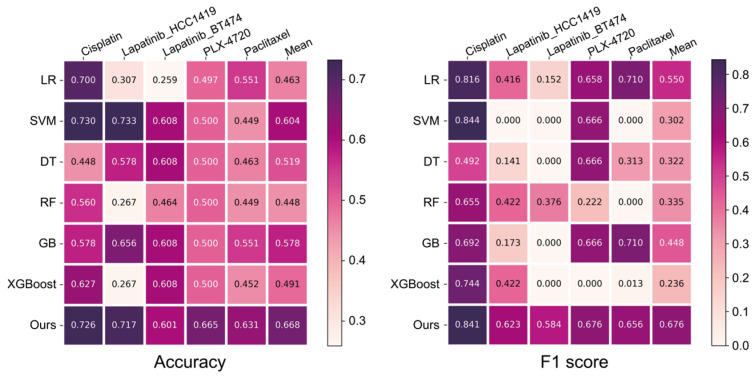
Comparison of prediction performance in five scRNA-seq drug response datasets.

As shown in Figure 3, our model achieved the best performance across the four scRNA-seq drug response prediction tasks. It reached an average accuracy of 0.668 and an average F1 score of 0.676, outperformed LR (0.463 and 0.550), SVM (0.604 and 0.302), DT (0.519 and 0.322), RF (0.448 and 0.335), GB (0.578 and 0.448), and XGBoost (0.491 and 0.236). Although the SVM model slightly outperformed our method in predicting drug response for Cisplatin, the performance gap was marginal (accuracy of 0.730 vs. 0.726, F1 score of 0.844 vs. 0.841) and our model outperformed all other ML models on predicting drug response for Cisplatin. Moreover, our model achieved superior results on the remaining three drugs compared to all ML models. Notably, five ML models yielded an F1 score of 0 in at least one task, particularly on the Lapatinib_BT474 and Paclitaxel datasets. In contrast, our model maintained consistently high performance across all tasks. This further demonstrates the robustness of our model, especially in handling complex scRNA-seq data and imbalanced datasets.

### 2.3. Impact of Key Hyperparameters on Model Performance

To optimize the predictive performance of our model, we tuned several key hyperparameters that had a significant impact on model performance. Notably, since both the Lapatinib_BT474 and Lapatinib_HCC1419 datasets involve the same drug, they were merged to investigate how individual hyperparameters affect the model’s performance in predicting drug responses for Lapatinib.

**Figure 4:**
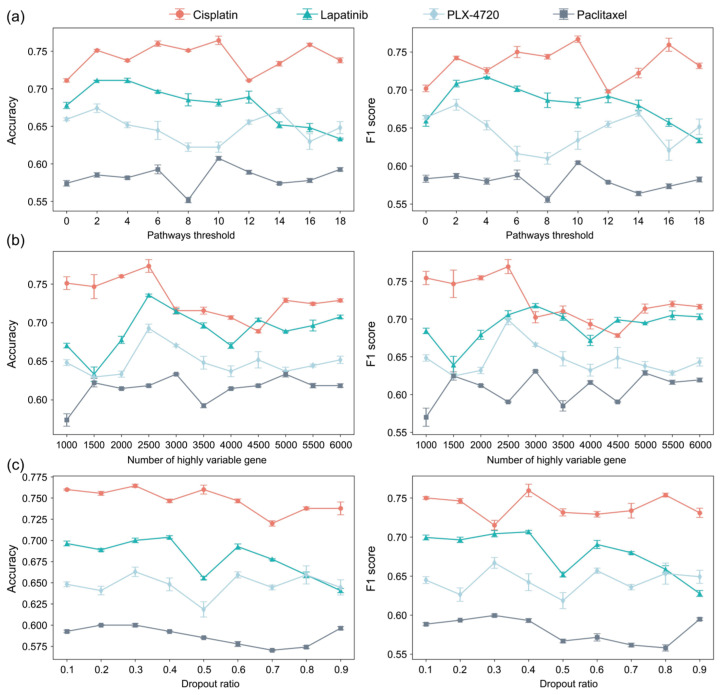
The effect of three hyperparameters on model performance. (**a**) The effect of pathways threshold; (**b**) the effect of the number of highly variable genes; (**c**) the effect of dropout ratio.

For a given pathway, only a specific subset of genes is typically expressed. As a result, the corresponding row in the mask matrix of the sparse decoder is dominated by 0. To prevent the decoder from becoming excessively sparse, which may impair its ability to learn meaningful representations and to capture pathway-level prior biological knowledge, we applied a threshold to exclude pathways with an insufficient number of associated genes. Figure 4a presents the model’s validation performance across multiple datasets using Gene Ontology (GO) [34] pathway data under various pathways thresholds.

Given the limited size of each classification training dataset, we only randomly selected 10% of the samples as a validation set for hyperparameter tuning. Each experiment was repeated five times with different random seeds, and the average results were calculated.

The results show that for the Cisplatin and Paclitaxel datasets, the best predictive performance was achieved when the threshold was set to 10. In contrast, the PLX-4720 dataset performed better with a threshold of 2. For the Lapatinib dataset, although thresholds of 2 and 4 both yielded accuracy values above 0.7, the model achieved a higher F1 score when the threshold was set to 4.

Other hyperparameters that influenced model performance included the number of highly variable genes (HVGs), and the dropout ratio used in the neural network. The effects of these two hyperparameters on model performance are shown in Figure 4a,b.

For the number of HVG, we tested several values and observed that the optimal setting varied slightly across datasets. Specifically, for the Paclitaxel dataset, the model achieved better accuracy and F1 score when HVG was set to 3000. For the other three drugs (Cisplatin, PLX-4720, and Lapatinib), the best validation performance was consistently observed when the number of HVG was set to 2500. This setting provided a favorable trade-off between retaining informative gene expression signals and avoiding overfitting due to noise or redundant features.

As for the dropout rate, we evaluated values ranging from 0.1 to 0.9. The model demonstrated robust performance when the dropout rate was set between 0.1 and 0.4. These results suggest that moderate dropout regularization was beneficial for enhancing the generalization ability of the models.

In addition, we also compared the different pathway information in constructing the sparse decoder. Commonly used biological pathway resources include GO [34], Reactome [35], and Hallmark [36]. GO encompasses all levels of biological systems, from molecular activities to complex cellular and organismal-level networks, offering reliable information of pathways. Reactome is a manually curated database of metabolic and pathways, while Hallmark contains 50 curated, non-redundant gene sets specifically designed for gene set enrichment analysis.

We evaluated the predictive performance of our model with sparse decoders constructed from this three different pathway information on the test set GSE117872. For all pathway information, the minimum gene count threshold was fixed at 10. As shown in Table 2, the results indicated that the decoder based on the pathways information from GO achieved the best predictive performance among the three, demonstrating its suitability for modeling scRNA-seq drug response in this context.

### 2.4. Analysis of Modeling Strategies

To evaluate the importance of each module in the proposed model and its contribution to predictive performance, we conducted an ablation study and compared the effects of different strategies for aligning data across sequencing platforms on model performance.

The comparison includes a baseline model consisting of a naive autoencoder without transfer learning (base AE) followed by a multilayer perceptron classifier. To explore the impact of transfer learning, we further examined a previously proposed approach based on autoencoder integrated with generative adversarial networks (adv AE), which was originally proposed to align transcriptomic data between in vitro cell lines and in vivo clinical samples. Beyond these basic components, we further examined the performance of base AE and adv AE when combined with a shared encoder module (share AE). Finally, we assessed the full model (ours), which integrates the shared encoder with a pathway guidance (share AE + pathways).

The experimental results on the test set GSE117872 are summarized in Table 3. We observed that the incorporation of generative adversarial networks and a shared encoder significantly improved model performance. Our model, which integrated a sparse decoder to introduce pathway-level prior biological knowledge, outperformed other methods and achieved substantial improvements over the base AE. These results demonstrate that incorporating transfer learning strategies notably enhances performance. Furthermore, the addition of pathway-level guidance not only improves model interpretability but also contributes to improved accuracy.

**Figure 5:**
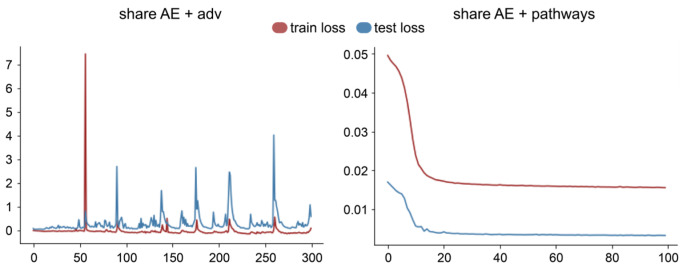
Comparison of loss between models integrating adversarial strategy and pathway into shared encoder.

As shown in Figure 5, we also observed that introducing a generative adversarial strategy into the model made training more difficult and increased the risk of overfitting. This observation contrasts with the findings reported in CODE-AE-ADV, where a generative adversarial strategy is effectively applied to align in vitro and in vivo data.

### 2.5. Pathways Attribution

Using the test set Lapatinib_BT474 as an example, we visualized the prediction results and conducted an in-depth interpretability analysis with an external interpretability algorithm to analyze the latent embeddings generated by autoencoder, which is incorporated with pathway information.

**Figure 6:**
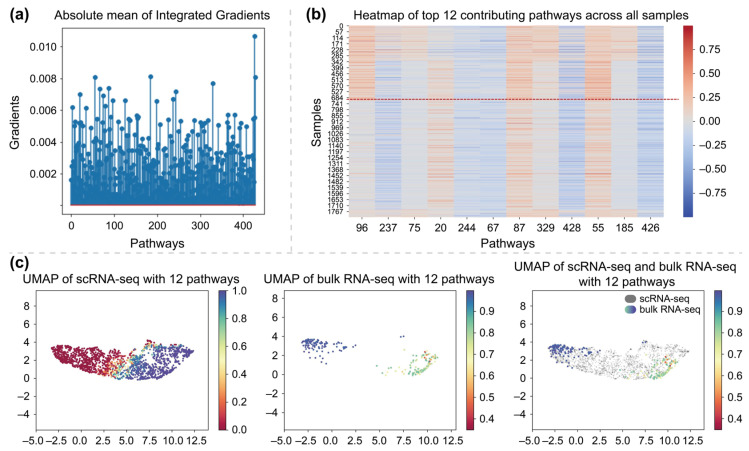
Presents three aspects of the interpretability analysis. (**a**) Results of integrated gradients for pathways; (**b**) heatmap of 12 most contributing pathways; (**c**) UMAP for both of scRNA-seq and bulk RNA-seq.

Specifically, we employed IG [37] to quantify the contribution of each pathway to the drug response prediction. IG is an interpretability technique that aims to attribute model’s predictions to its input features and is widely used to assess feature importance in DL models [38,39,40]. In our implementation, we calculated the mean absolute of IG values for each pathway to evaluate the influences of pathways on the prediction. A value close to 0 indicates less influence on the final prediction, while a higher absolute value reflects a stronger contribution. The results of IG were shown in Figure 6a. Based on this analysis, we identified the top 12 most influential features (i.e., those with the largest absolute IG values), which correspond to the pathways most relevant to the drug-response prediction, as illustrated in Figure 6b. Among these 12 pathways, 10 were predefined pathways based on prior biological knowledge, while the remaining 2 were additional dimensions specifically introduced in model to capture auxiliary pathway information. Of the 12 selected pathways, 7 exhibited positive contributions, aligning with predictions of sensitive cells, while 5 showed negative contributions across most samples, potentially indicating association with drug resistance mechanisms. Also, as shown in Figure 6c, UMAP visualization based on the top 12 pathways demonstrated that both of scRNA-seq and bulk RNA-seq were projected into the same low-dimensional space with similar distributions, indicating a high degree of consistency. This further supports the effectiveness of the selected top 12 pathways.

The contributions of these 12 pathways to individual samples were visualized in Figure 6b. It can be observed that each pathway exhibits relatively consistent attribution values across all samples. Notably, pathways 96, 75, 329, and 185 showed attribution patterns that aligned with the ground truth drug response labels. These pathways contributed positively to the predictions of sensitive cells, while their contributions to resistant cells remained close to 0. In contrast, pathways 244, 67, 428, and 426 exhibited predominantly negative contributions across nearly all samples, regardless of their actual response labels, suggesting a potential suppressive role in the prediction process.

To further interpret the learned features, we analyzed 10 predefined pathways by examining their associated GO terms. Notably, the 96th dimension (ranked first by IG) and the 185th dimension (ranked eleventh by IG) were associated with GOCC CASPASE COMPLEX and GOCC CULLIN RING UBIQUITIN LIGASE COMPLEX, respectively. These pathways are related to drug sensitivity mechanisms. The former is a protein complex that contains one or more cysteine-type endopeptidases, which may be involved in apoptotic processes. The latter is part of a protein degradation pathway mediated by the Cullin-RING ubiquitin ligase complex [41,42]. The high attribution scores of these pathways suggest that the model captured the potential association between drug sensitivity and GO pathways information during training.

We also identified four dimensions associated with pathways involved in drug resistance mechanisms. The 237th dimension (ranked second by IG) and the 55th dimension (ranked tenth) correspond to GOCC CALCIUM CHANNEL COMPLEX and GOCC ENDOPLASMIC RETICULUM LUMEN, respectively, both of which are linked to metabolic stress and cellular stress responses. The former is an ion channel complex through calcium ions pass, while the latter is the volume enclosed by the membranes of the endoplasmic reticulum. In addition, the 75th dimension (ranked third) and the 67th dimension (ranked sixth) are associated with GOCC MICROTUBULE and GOCC MICROTUBULE ORGANIZING CENTER, respectively, which are related to cytoskeletal dynamics and cell cycle arrest. The former is the microtubule, while the latter is the microtubule-organizing center, both of which are part of the cytoskeleton of eukaryotic cells [41,42]. These pathway associations further support the model’s ability to learn potential features and knowledge of drug resistance through GO pathways information.

## 3. Discussion

In this study, we collected and curated scRNA-seq drug response datasets. Five datasets were selected for testing, which cover three cancers and four cancer-related drugs. The DL clustering model, AttentionAE-sc, was applied to process the collected data. The clustering results were utilized to exclude normal cells and cells in the control group that exhibit genetic resistance. In addition, high-quality labeling was achieved by incorporating prior biological knowledge derived from published literature. To validate the effectiveness of AttentionAE-sc, we analyzed its clustering results, which yielded ASW scores above 0.75 across all datasets, indicating highly reliable clustering performance.

We utilized these five scRNA-seq datasets as testing data and their corresponding bulk RNA-seq data from the GDSC as training data to construct a drug response predictive model. Compared with ML models, the proposed DL model demonstrated superior performance across five drug response datasets. Our model attained an average accuracy of 0.668 and an average F1 score of 0.676. In comparison, the worst-performing ML model in accuracy was LR, which achieved 0.463 for accuracy and 0.550 for F1 score. Our model outperformed it by 0.205 and 0.126, respectively. The best-performing ML model in accuracy was SVM, which achieved 0.604 and 0.302 in accuracy and F1 score, our model showed improvements of 0.064 and 0.374. Regarding F1 score, the worst-performing ML model was XGBoost, with 0.491 for accuracy and 0.236 for F1 score. Our model surpassed it by 0.177 and 0.440, respectively. Compared to the best-performing model in F1 score, LR (0.463 and 0.550), our model achieved notable gains of 0.205 in accuracy and 0.126 in F1 score. The SVM model slightly outperformed our method in predicting drug response for Cisplatin, which may be attributed to the relative simplicity of this dataset (the feature differences between positive and negative samples are pronounced). As a result, all models achieved strong performance on this dataset. Since SVMs are designed to separate samples using an optimal hyperplane, they tend to excel on binary classification tasks with clear class boundaries and their slight advantage is unsurprising.

Our model exhibited relatively consistent accuracy and F1 scores across all datasets, suggesting improved stability and a stronger ability to capture latent patterns in gene expression related to drug response that are not well detected by ML models. Meanwhile, we observed that several ML models yielded an F1 score of 0 on some datasets. Subsequent analysis revealed that this was caused by a recall value of 0, suggesting that these models classified all samples as negative (resistant). This outcome reflects a failure to learn the underlying associations between transcriptomic features and drug response. The recurrence of such issues across multiple ML models further highlights the advantages of DL in accurately predicting drug response and its ability to interpret latent biological features and relationships.

We developed a shared autoencoder framework incorporating biological pathway information to explore the potential of transfer learning between bulk RNA-seq and scRNA-seq drug response datasets. We found that introducing pathway-level prior biological knowledge and employing a transfer learning strategy significantly enhanced model performance. Notably, Gene Ontology (GO) pathways provided the most reliable source of biological knowledge in our experiments. Moreover, ablation experiments revealed that the inclusion of generative adversarial strategies made the model more challenging to optimize and increase the risk of overfitting. This observation contrasts with previous findings using adversarial strategies on in vitro and in vivo bulk RNA-seq data. We hypothesize that this discrepancy may arise from the larger distributional differences between scRNA-seq and bulk RNA-seq data. Specifically, scRNA-seq data typically exhibit lower total gene expression counts and higher sparsity than bulk RNA-seq data, which may demand larger training sample sizes to ensure effective adversarial optimization.

To assess the interpretability of our model, we employed IG to interpret and visualize the association between the pathway knowledge and the predicted drug response outcomes. The results indicated that integrating biological knowledge improved both the reliability and interpretability of the model. Based on IG values, we identified the top 12 pathways that had a significant impact on the predictions. Among them, we conducted detailed analysis on 10 predefined pathways of these identified pathways. The literature review revealed that two of them were associated with drug sensitivity mechanisms, while four were related to drug resistance mechanisms.

The two pathways associated with drug sensitivity were the caspase complex-mediated apoptotic pathway and the Cullin-RING ubiquitin ligase complex-mediated protein degradation pathway. Lapatinib, as a dual HER2/EGFR tyrosinase inhibitor, may competitively bind the intracellular kinase domain and selectively block HER2 downstream pathways including PI3K/AKT and MAPK, while simultaneously triggering caspase-3/9 cascade activation to induce mitochondrial-dependent apoptosis. Therefore, in HER2-overexpressing BT474 cell lines, Lapatinib may disrupt the balance between proliferation and apoptosis, shifting cells toward a drug-sensitive state. The Cullin-RING complex may also contribute to Lapatinib sensitivity by mediating degradation of prosurvival proteins [43,44].

Among the drug resistance-related pathways, two were associated with metabolic stress and cellular stress responses (calcium channel complex and endoplasmic reticulum lumen), while the other two were related to cytoskeletal dynamics and cell cycle arrest (microtubule and microtubule organizing center). Lapatinib may inhibit downstream signaling of HER2, including the PI3K/AKT pathway, resulting in decreased endoplasmic reticulum calcium pump activity. This may trigger endoplasmic reticulum stress and activate the unfolded protein response. Resistant cells may counteract this by upregulating CD36-mediated lipid uptake, which alleviates oxidative stress in endoplasmic reticulum membrane lipid. Additionally, enhanced endoplasmic reticulum associated degradation of misfolded proteins may contribute to the maintenance of proteostasis, enabling cells to withstand drug-induced stress [45,46]. Regarding the cytoskeletal-related mechanisms, HER2 signaling regulates microtubule dynamics and cell division. Lapatinib interferes with the interaction between HER2/EGFR and microtubule-associated proteins, disrupting the assembly of γ-tubulin ring complexes and leading to abnormal mitotic spindle formation. In treated cells, metabolic plasticity may enhance ATP production to maintain the activity of microtubule, thereby mitigating cytoskeletal damage induced by Lapatinib. This may potentially contribute to drug resistance [47,48].

However, our model was limited to using only gene expression data without integrating drug-specific features or biological context information (such as cancer type). This constraint limits the model’s capacity for pan-drug and pan-cancer generalization. In future work, we plan to incorporate more information, such as drug molecular structures, cancer types, and tissue-specific characteristics, with multimodal learning to enhance prediction performance across diverse drugs and cancer types. Moreover, the continued expansion of available scRNA-seq drug response datasets will support the development of a more generalized and robust predictive framework.

## 4. Materials and Methods

### 4.1. Data Collection and Processing

#### 4.1.1. Collection

The Gene Expression Omnibus (GEO, https://www.ncbi.nlm.nih.gov/geo/, accessed on 1 November 2024) [49], a public functional genomics data repository maintained by National Center for Biotechnology Information (NCBI), contains high-throughput transcriptomic data submitted by institutions worldwide. In this study, we conducted retrieval and filter based on the following keywords and criteria: based on scRNA-seq platforms; inclusion of control group; exclusion of radiotherapy; exclusion of combination therapy; and drug-treated experimental groups receiving doses and treatment durations sufficient to induce drug resistance.

We retrieved corresponding drug response data for these datasets from the GDSC database (bulk RNA-seq) based on matched drugs and cell lines. Both bulk RNA-seq and scRNA-seq data were used during the pretraining phase to train the autoencoder, aiming to obtain a shared encoder to map both data types into a unified latent space. Subsequently, during the classifier training phase for drug response prediction, bulk RNA-seq data were used as the training and validation sets, while scRNA-seq datasets served as the test set. The classifier trained on bulk RNA-seq was directly transferred to predict on single-cell data to fully leverage the abundance of bulk data and compensate for the current scarcity of scRNA-seq drug response data.

#### 4.1.2. Labeling Strategy for Drug Response

Previous studies on scRNA-seq drug response have typically labeled drug-treated groups as resistant and control group as sensitive. However, due to the presence of genetic resistance to drugs, the control group may contain subpopulations that are intrinsically insensitive to the drug. These cells need to be identified and excluded. Moreover, most in vivo experimental datasets include highly differentiated normal cells, which should also be filtered out.

To address these issues, we proposed an improved labeling strategy. Firstly, we collected individual scRNA-seq drug response datasets. Then, we performed clustering analysis using AttentionAE-sc model to identify cell clusters corresponding to resistant or sensitive states. Based on the clustering results, cells in the control group that were likely to be drug-insensitive were excluded. Subsequently, we incorporated prior biological knowledge from public resources, such as marker gene sets, to filter out highly differentiated normal cells. Furthermore, the literature was used to assign accurate drug response labels to tumor cells.

We applied AttentionAE-sc model for clustering analysis with following parameters: 2500 highly variable genes selected, eight attention heads, cell embedding dimension of 16, Leiden resolution of 0.1, and a Gaussian kernel for graph construction. The number of cells used for the model was limited to 4000. Data preprocessing followed the original pipeline, implemented using the Scanpy toolkit. In our implementation, this parameter set achieved consistently high ASW scores and yielded optimal clustering performance. However, if significantly different parameters were used, especially those resulting in a substantial decrease in clustering quality, the robustness of the exclusion step could be compromised, and downstream predictions might be adversely affected. Therefore, careful parameter tuning remains a critical consideration when applying AttentionAE-sc in similar tasks.

The detailed labeling procedure involved the following steps. First, cells in the control group that exhibited transcriptional profiles similar to those of the drug-treated group were considered having genetic resistance. Subsequently, they were excluded from the sensitive (positive) sample set. Second, subpopulations corresponding to normal cells were identified based on known marker genes and then excluded. Finally, for certain targeted therapies, drug response labels were then assigned based on literature-reported differences in drug sensitivity among the identified tumor subpopulations.

### 4.2. Shared Encoder for Different Sequencing Data

CODE-AE [19] was trained simultaneously on both in vitro and in vivo data, yielding a deconfounding shared encoder that aligns these two data in the latent space. Inspired by this method, we aim to leverage a shared encoder to align bulk RNA-seq and scRNA-seq data. Therefore, we adapted the architectural design and training strategies of CODE-AE. The autoencoder is composed of two encoders and a decoder. The two encoders are designed to separately process bulk and single-cell RNA-seq data, and can be described as follows:

(1) $$\left\{\begin{array}{c} Z_{sc-p}=ReLU(X_{sc}\cdot W_{sc-p^{1}}+b_{1})\cdot W_{sc-p^{2}}+b_{2}\\ Z_{bulk-p}=ReLU\left(X_{bulk}\cdot W_{bulk-p^{1}}+b_{1}\right)\cdot W_{bulk-p^{2}}+b_{2} \end{array}\right.$$

(2) $$\left\{\begin{array}{c} Z_{sc-s}=ReLU(X_{sc}\cdot W_{s^{1}}+b_{1})\cdot W_{s^{2}}+b_{2}\\ Z_{bulk-s}=ReLU(X_{bulk}\cdot W_{s^{1}}+b_{1})\cdot W_{s^{2}}+b_{2\;} \end{array}\right.$$

where Z represents the output tensors of the encoder. All the sequencing data have two output tensors: $Z_{s}$ of the shared encoder and $Z_{p}$ of the private encoder, $X_{bulk}$ and $X_{sc}$ refer to the bulk RNA-seq and scRNA-seq data. W and *b* are learnable parameters, where W represents weights and *b* represents biases. $W_{s}$ refer to the shared weight of the shared encoder and is shared across both sequencing data, while $W_{p}$ represent the weights of the encoders separately applied to bulk and single-cell RNA-seq data. The two layers of W indicate that the encoders consist of two fully connected layers. The activation function used in the encoder is ReLU, which is defined as follows:

(3) $$x=\left\{\begin{array}{c} x,\;if\;x>0\\ 0,\;if\;x\;\leq 0 \end{array}\right.$$

In the decoder, the two output tensors for each dataset are concatenated and passed through a decoding layer with the same structure as the encoder to reconstruct the gene expression matrices:

(4) $$\left\{\begin{array}{c} \bar{X_{sc}}=ReLU(concat\left[Z_{sc-p},\;Z_{sc-s}\right]\cdot W_{dec^{1}}+b_{1})\cdot W_{dec^{2}}+b_{2}\\ \bar{X_{bulk}}=ReLU(concat\left[Z_{bulk-p},\;Z_{bulk-s}\right]\cdot W_{dec^{1}}+b_{1})\cdot W_{dec^{2}}+b_{2} \end{array}\right.$$

The reconstruction loss of the autoencoder is computed as the sum of the reconstruction losses for both datasets, using the mean squared error (MSE) with the L2 norm:

(5) $$L_{recon}=\|X_{sc}-\bar{X_{sc}}{\|}^{2}+\|X_{bulk}-\bar{X_{bulk}}{\|}^{2}$$

To guide two encoders to learn distinct features (shared and private features), we introduce an orthogonality constraint to minimize redundancy between the two embeddings:

(6) $$L_{diff}=\|Z_{sc-p}\cdot Z_{sc-s}{\|}^{2}+\|Z_{bulk-p}\cdot Z_{bulk-s}{\|}^{2}$$

Thus, the final autoencoder loss function is defined as follows:

(7) $$L_{AE}=L_{recon}+r_{1}\;L_{diff}$$

where $r_{1}$ is a balancing factor for multi-objective optimization, with a default value of 0.1.

### 4.3. Incorporating Biological Information with Sparse Decoder

To enhance the interpretability of the autoencoder, a sparse decoder was introduced to constrain the shared encoder. Similarly to VNNs, which incorporate prior biological knowledge through sparsely connected decoding structures [50,51], the sparse decoder in our model introduce prior biological knowledge based on predefined pathway–gene associations. And the sparse decoder can be defined as follows:

(8) $$\bar{X_{sc^{sparse}}}=ReLU(Z_{sc-s}\cdot W_{sparse}+b)$$

(9) $$\bar{X_{bulk^{sparse}}}=ReLU(Z_{bulk-s}\cdot W_{sparse}+b)$$

(10) $$L_{sparse}=\|X_{sc}-\bar{X_{sc^{sparse}}}{\|}^{2}+\|X_{bulk}-\bar{X_{bulk^{sparse}}}{\|}^{2}$$

where $W_{sparse}$ represents the sparse weight matrix with a mask, defined as $W_{sparse}=W\cdot M_{mask}$. The mask matrix $M_{mask}$ is constructed based on prior biological pathway information, where each row represents a biological pathway, and each column represents a gene. $M_{mask}$ consists of 1 and 0, where 1 indicates that the gene is a regulatory gene of the pathway, and 0 indicates that the gene is not associated with the pathway. To allow the model to capture information beyond the predefined biological pathways, five additional rows consisting entirely of 1 are concatenated to the mask matrix. This enables the encoder to learn latent biological patterns that are not constrained by existing predefined pathways. The output tensor Z of the encoder has a dimension equal to the number of rows in the mask matrix $M_{mask}$. Apart from the five additional dimensions, each dimension in the latent space corresponds to a gene associated with a specific pathway.

The optimization objective for training is defined as follows:

(11) $$L_{train}=L_{AE}+r_{2}\;L_{sparse}$$

where $r_{2}$ is a balancing factor for multi-objective optimization, with a default value of 0.1.

After the autoencoder is trained, the shared encoder is used to project data from the two different sequencing platforms into a unified latent space. The resulting embeddings then serve as inputs for the drug response prediction model in both the training and inference stages.

### 4.4. Labels of Dataset and Training for Classifier

Label information for drug response of cell lines is required for training a predictive model of drug response. The drug response information in GDSC database includes two labels: IC50, where a lower value indicates stronger drug efficacy and higher sensitivity and AUC, where a lower value indicates greater sensitivity to treatment). Both of them are continuous, which need to be converted to discrete for training a classification model. To achieve this, we adopted an extreme value selection strategy to discretize the labels. Specifically, for each drug, cell lines were ranked based on AUC values, and a threshold parameter *k* (0 < *k* < 0.5) was defined. The top *k* fraction of cell lines with the lowest AUC values was labeled as sensitive (y = 1), while the top *k* fraction with the highest AUC values was labeled as resistant (y = 0). The drug response prediction model is constructed with a three-layer neural network classifier, as follows:

(12) $$H=ReLU(ReLU(Z\cdot W_{1}+b_{1})\cdot W_{2}+b_{2})$$

(13) $$y_{pred}=Sigmoid(H\cdot W+b)$$

where the first two layers use the ReLU as the activation function, and H represents the output tensor of the second layer. The final layer employs the Sigmoid activation function to map the output to the range [0, 1], which is defined as follows:

(14) $$Sigmoid\left(x\right)=\frac{1}{1+e^{-x}}$$

The cross-entropy loss was used for training:

(15) $$L=-\frac{1}{N}\overset{N}{\underset{i=1}{\sum }}\left[y_{i}log\left(y_{pred^{i}}\right)+\left(1-y_{i}\right)\log\left(1-y_{pred^{i}}\right)\right]$$

where $y_{i}$ represents the label of the i-th sample, $y_{pred^{i}}$ represents the predicted probability for the i-th sample, and N is the total number of samples.

### 4.5. Model Performance Evaluation Metrics

We adopted accuracy and F1 scores to evaluate the predictive performance of the classification model. Accuracy measures the overall correctness of predictions and serves as a general indicator of model performance. However, in imbalanced datasets (the number of positive and negative samples differs significantly) accuracy alone may not adequately reflect the model’s ability to correctly identify the minority class. To address this limitation, we utilized the F1 score, which considers both precision and recall. Precision is defined as the proportion of true positive predictions among all samples predicted as positive, while recall represents the proportion of true positives that are correctly identified among all actual positive samples. The F1 score is the harmonic mean of precision and recall; it penalizes models heavily when either precision or recall is low, making it particularly suitable for evaluating models on imbalanced datasets. These metrics are defined as follows:

(16) $$\mathrm{Accuracy}=\frac{\mathrm{TP}+\mathrm{TN}}{\mathrm{TP}+\mathrm{TN}+\mathrm{FP}+\mathrm{FN}}$$

(17) $$\mathrm{precision}=\frac{\mathrm{TP}}{\mathrm{TP}+\mathrm{FP}}$$

(18) $$\mathrm{recall}\;\mathrm{rate}=\frac{\mathrm{TP}}{\mathrm{TP}+\mathrm{FN}}$$

(19) $$F1\;\mathrm{score}=\frac{2\times \left(\mathrm{precision}\times \mathrm{recall}\;\mathrm{rate}\right)}{\mathrm{precision}+\mathrm{recall}\;\mathrm{rate}}=\frac{2\mathrm{TP}}{2\mathrm{TP}+\mathrm{FP}+\mathrm{FN}}$$

TP (true positives) refers to the number of samples that are correctly predicted as positive and are indeed positive. TN (true negatives) refers to the number of samples that are correctly predicted as negative and are indeed negative. In contrast, FP (false positives) denotes the number of samples whose true label is negative but are incorrectly predicted as positive, and FN (false negatives) denotes the number of samples whose true label is positive but are incorrectly predicted as negative.

## 5. Conclusions

In conclusion, this study systematically collected and curated scRNA-seq drug response datasets and proposed a robust and interpretable DL model for predicting drug responses in scRNA-seq by leveraging transfer learning from bulk RNA-seq. These advances may contribute to the development of precision medicine and enhance understanding of mechanisms in cancer. However, our model is limited to single-drug prediction, primarily due to the scarcity of available data. Future work will aim to expand the diversity and scale of annotated datasets and explore multimodal learning approaches by incorporating additional features, such as drug structures. These efforts may further enable the development of predicting responses to multiple drugs and drug combinations using scRNA-seq data.

## Figures and Tables

**Table 1:** Summary of scRNA-seq drug response datasets and corresponding data in GDSC.

Dataset	Drug	Number of Sensitive/ Resistant Cells	Number of Cells in GDSC
GSE117872	Cisplatin	950/352	735
GSE131984	Paclitaxel	922/752	895
GSE108394	PLX-4720	3242/3236	898
GSE156246_BT474	Lapatinib	714/1107	903
GSE156246_HCC1419	Lapatinib	1584/4346	903

scRNA-seq: Single-cell RNA sequencing; GDSC: Genomics of Drug Sensitivity in Cancer.

**Table 2:** Performance of three different pathways information resources.

Pathways Information Resource	Accuracy	F1 Score
Gene Ontology	0.726 ± 0.008	0.841 ± 0.007
Reactome	0.710 ± 0.008	0.825 ± 0.007
Hallmark	0.696 ± 0.011	0.815 ± 0.009

**Table 3:** Performance of different modeling strategies.

Methods	Accuracy	F1 Score
base AE	0.2976	0.0834
adv AE	0.4545	0.3393
base share AE	0.3538	0.2407
adv share AE	0.5487	0.5145
ours (share AE + pathways)	0.7130	0.8308

## Data Availability

All data used in this study were originally obtained from Gene Expression Omnibus (GEO, https://www.ncbi.nlm.nih.gov/geo/, accessed on 1 November 2024) and Genomics of Drug Sensitivity in Cancer (GDSC, https://www.cancerrxgene.org/, accessed on 1 December 2024). The collected and curated datasets are available on Figshare (10.6084/m9.figshare.28888880). The model code is available on GitHub (https://github.com/FEIFEIEIAr/bulk2single-cell, accessed on 1 November 2024).

## Supplementary Materials

The following supporting information can be downloaded at: https://www.mdpi.com/article/10.3390/ijms26094365/s1. References [52,53] are cited in the Supplementary Materials.

## Abbreviations

The following abbreviations are used in this manuscript:

scRNA-seq	Single-cell RNA sequencing
DL	Deep learning
ML	Machine learning
RF	Random forest
VNN	Visible neural network
bulk RNA-seq	Bulk RNA sequencing
GDSC	Genomics of Drug Sensitivity in Cancer
IG	Integrated gradients
IC50	Half maximal inhibitory concentration
AUC	The area under the dose–response curve
ASW	Average silhouette width
UMAP	Uniform Manifold Approximation and Projection
LR	Logistic regression
SVM	Support vector machine
DT	Decision tree
RF	Random forest
GB	Gradient boosting
XGBoost	eXtreme Gradient Boosting
GO	Gene ontology
HVG	Highly variable gene
GEO	Gene Expression Omnibus
