# Supplementary material for: A Transfer Learning Framework for Predicting and Interpreting Drug Responses via Single-Cell RNA-Seq Data
Source: Int J Mol Sci. 2025 May 4;26(9):4365. doi: 10.3390/ijms26094365 (PMC12072357; doi:10.3390/ijms26094365)
Supplement: Supplementary file 1 [file ijms-26-04365-s001.zip › ijms-3614586-supplementary.pdf]

International Journal of
Molecular Sciences

MDPI

Article

Supplementary Materials

S1. Data Collection

As shown in Table S1, a total of 23 scRNA-seq drug response datasets were collected and curated, comprising 18 human-derived datasets and 5 mouse-derived datasets. These datasets cover breast cancer (8 datasets), prostate cancer (3 datasets), lung cancer (3 datasets), Squamous cell carcinoma (3 datasets), melanoma (2 datasets), leukemia (2 datasets), and one dataset each for the remaining cancer types. The datasets involve a diverse range of therapeutic agents, including 3 broad-spectrum chemotherapeutic drugs (paclitaxel, cisplatin, homoharringtonine (HHT)), 3 immunotherapeutic agents (IL1B, nivolumab, and NP137), and 11 targeted therapies.

As shown in Figure S1, the clustering results for all datasets demonstrated good performance. Specifically, 8 datasets achieved an average silhouette width (ASW) greater than 0.8, and 19 datasets achieved an ASW greater than 0.7. Only one dataset had an ASW below 0.65, and this dataset was not included in subsequent model development.

It is important to note that only those datasets listed in Table 1 had corresponding entries in the GDSC database. Therefore, only these five datasets were utilized for subsequent model construction.

Table S1. Summary of collected scRNA-seq drug response datasets. GEO: Gene Expression Omnibus.

GEO ID	Drug	Sample Description	Number of Cells
GSE108394	PLX-4720	Melanoma cell line 451Lu	6545
GSE129730	Vismodegib	Mouse medulloblastoma	36841
GSE131984	Paclitaxel	Breast cancer cell line SUM159	1813
GSE131984	Palbociclib	Breast cancer cell line SUM159	1754
GSE156246	Lapatinib	Breast cancer cell line BT474	3321
GSE156246	Lapatinib	Breast cancer cell line HCC1419	15052
GSE161741	Cisplatin	Mouse small-cell lung cancer (SCLC) cell line	7980
GSE164897	Vemurafenib	Melanoma cell line A375	11023
GSE168668	Enzalutamide	Prostate cancer cell line LNCaP	17233
GSE175975	Enzalutamide	Prostate cancer cell line sgNT	32349
GSE175975	Enzalutamide	Prostate cancer cell line with TP53, RB1, JAK1 knockout	39653
GSE117872	Cisplatin	Primary oral squamous cell carcinoma cells from patients	1302
GSE195832	Nivolumab	Patient-derived head and neck squamous cell carcinoma cells	59205
GSE201664	AG120	Mouse model of AML (IDH1 mutation)	8162
GSE229067	Sotorasib	Lung cancer cell line H23	24066
GSE229067	Sotorasib	Lung cancer cell line H358	20892
GSE234265	NP137	Mouse primary cutaneous squamous cell carcinoma	57011
GSE237848	IL1B	Mouse pancreatic ductal adenocarcinoma	16400
GSE252745	HHT	Patient-derived acute myeloid leukemia cells	6597
GSE274729	DHT	Breast cancer cell line T47D	10881
GSE274729	Enobosarm	Breast cancer cell line T47D	12952
GSE274729	DHT	Breast cancer patient-derived xenograft (PDX, WHIM23)	3152
GSE274729	Enobosarm	Breast cancer patient-derived xenograft (PDX, WHIM23)	4567

 https://doi.org/10.3390/xxxxx

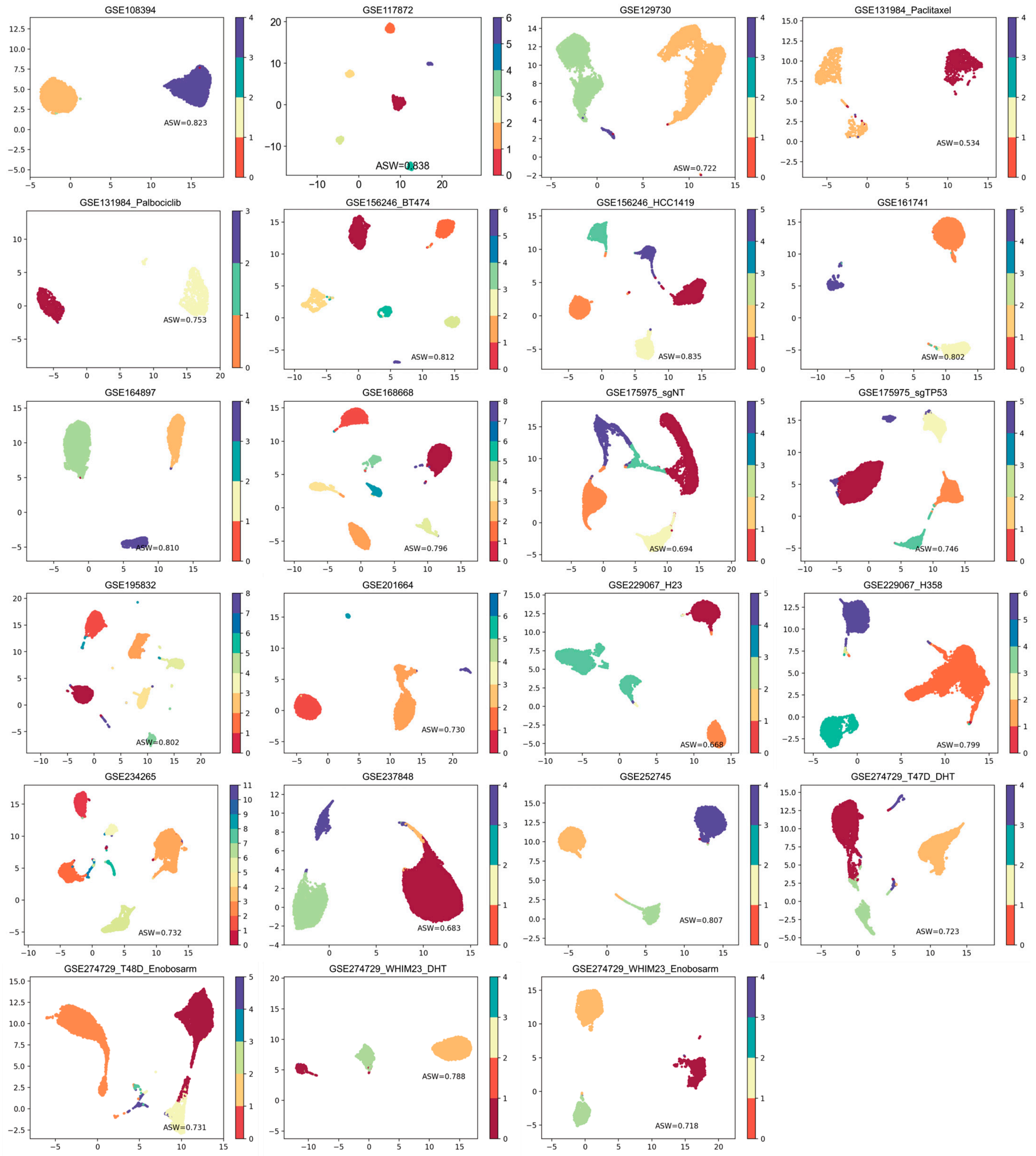

Figure S1. UMAP visualization of clustering results for all collected scRNA-seq drug response datasets. UMAP: Uniform Manifold Approximation and Projection; ASW: Average silhouette width.

S2. Validation in Clustering

To validate the robustness of the clustering results produced by AttentionAE-sc, we compared its performance against several other clustering methods. K-Means and Agglomerative Clustering are classical machine learning algorithms. K-Means partitions the

samples into *k* clusters by minimizing the distance between each sample and the nearest cluster center. Agglomerative Clustering is a bottom-up hierarchical clustering approach that successively merges pairs of clusters based on a defined similarity or distance metric. We also compared two deep learning-based approaches. scDeepCluster [1] is a deep embedded clustering method that simultaneously learns feature representations and performs clustering through explicit modeling of scRNA-seq data generation. scGNN [2] utilizes graph neural networks to learn feature embeddings from single-cell data.

As shown in Table S2, AttentionAE-sc outperformed all the other methods. Notably, K-Means yielded the poorest clustering performance, highlighting the difficulty of applying traditional ML methods to the highly complex and sparse nature of scRNA-seq data. In contrast, DL methods generally exhibited superior performance, underscoring their advantage in capturing intricate nonlinear relationships in single-cell data.

It is important that clustering quality directly impacts the reliability of subsequent data curation and labeling. Poor clustering results could lower confidence in the collected data and reduce the robustness of downstream analyses. In particular, unreliable clustering may introduce noise and bias into the training process, ultimately compromising the predictive performance of drug response models. Therefore, achieving high-quality clustering through AttentionAE-sc provides a solid and reliable foundation for the later stages of model construction and evaluation.

Table S2. Comparison of average silhouette width for clustering methods.

Dataset	AttentionAE-sc	K-Means	Agglomerative Clustering	scDeepCluster	scGNN
GSE117872	0.838	0.258	0.687	0.733	0.698
GSE131984	0.753	0.193	0.524	0.534	0.597
GSE108394	0.823	0.267	0.676	0.791	0.722
GSE156246_BT474	0.812	0.214	0.634	0.803	0.624
GSE156246_HCC1419	0.835	0.267	0.698	0.779	0.701

References

1. Tian, T.; Wan, J.; Song, Q.; Wei, Z. Clustering Single-Cell RNA-Seq Data with a Model-Based Deep Learning Approach. *Nat. Mach. Intell.* **2019**, *1*, 191–198, doi:10.1038/s42256-019-0037-0.

2. Wang, J.; Ma, A.; Chang, Y.; Gong, J.; Jiang, Y.; Qi, R.; Wang, C.; Fu, H.; Ma, Q.; Xu, D. scGNN Is a Novel Graph Neural Network Framework for Single-Cell RNA-Seq Analyses. *Nat. Commun.* **2021**, *12*, 1882, doi:10.1038/s41467-021-22197-x.
